# OA12 When steroids may not be the answer in polymyalgia rheumatica

**DOI:** 10.1093/rap/rkag113.012

**Published:** 2026-11-03

**Authors:** Alice Bowen, Jamie Robertson, Julia McEvoy, Lama Rawashdeh

**Affiliations:** Frimley Park Hospital, Surrey, United Kingdom; Frimley Park Hospital, Surrey, United Kingdom; Frimley Park Hospital, Surrey, United Kingdom; Frimley Park Hospital, Surrey, United Kingdom

## Abstract

**Introduction:**

Polymyalgia rheumatica (PMR) is a systemic inflammatory disease affecting approximately 1% of the United Kingdom population. It typically presents with inflammatory-sounding pain and stiffness in the hip and shoulder girdles, although its clinical spectrum is broad and diagnosis can be challenging due to numerous mimicking conditions. Diagnosis is primarily clinical, supported by elevated inflammatory markers, with imaging reserved for diagnostically uncertain cases. Glucocorticoids remain the cornerstone of treatment; however, 29–45% of patients do not respond adequately to initial therapy (1). Limited evidence exists for steroid-sparing agents. This case highlights the challenges of diagnosing and managing steroid-resistant PMR.

**Case description:**

A 56-year-old woman presented with a 3-month history of bilateral shoulder and hip pain and stiffness, most pronounced in the morning and associated with prolonged inactivity (gelling). She also reported malaise, flu-like symptoms, poor sleep, mild frontal headache, and unintentional weight loss. She denied rash, oral ulceration, Raynaud’s phenomenon, jaw claudication, or visual disturbance. Her medical history was unremarkable. She was a non-smoker, consumed alcohol within recommended limits, and had a family history of giant cell arteritis (GCA) in her mother.

Examination demonstrated restricted shoulder movement without synovitis, enthesitis, dactylitis, nail changes, rheumatoid nodules, or peripheral oedema.

Initial investigations showed a CRP of 59 mg/L, CK of 20 U/L, and negative ANA, ENA, rheumatoid factor, and anti-CCP antibodies. Chest radiography and temporal artery ultrasound were unremarkable.

A working diagnosis of PMR was made, and prednisolone 15 mg daily was commenced. However, her symptoms failed to improve, and CRP increased to 71 mg/L. Prednisolone was escalated to 15 mg twice daily and subsequently 40 mg daily. After three weeks, CRP had fallen to 22 mg/L, normalising to 4 mg/L after six weeks. Despite this biochemical response, she reported only modest symptomatic improvement.

Differential diagnoses included refractory PMR, large vessel vasculitis, inflammatory myopathy, and inflammatory arthritis. Following a steroid washout period, further imaging was performed. Whole-body MRI demonstrated no evidence of myositis. FDG PET-CT showed bilateral shoulder synovitis, bursitis, biceps tenosynovitis, and inflammation of the peri-articular fascial tissues, with no evidence of vasculitis or malignancy. These findings supported the diagnosis of PMR.

Prednisolone was restarted using a tapering regimen alongside methotrexate 15 mg weekly and hydroxychloroquine 200 mg daily. Despite combination therapy, symptom control remained suboptimal, raising concern for coexisting inflammatory arthritis and prompting consideration of biologic therapy, including anti-IL-6 treatment with Tocilizumab.

**Discussion:**

This case highlights the diagnostic and therapeutic challenges associated with PMR. PMR may present with non-specific symptoms that overlap with other rheumatic and non-rheumatic conditions. Elderly-onset rheumatoid arthritis, seronegative inflammatory arthritis, large vessel vasculitis, and other inflammatory disorders can closely mimic PMR, while the absence of disease-specific biomarkers further complicates diagnosis (2,3).

Imaging can be valuable in diagnostically challenging cases, in part to exclude alternative diagnoses. Ultrasound scanning of the shoulders can identify characteristic findings such as inflammation or effusion of the subacromial bursa, synovitis at the hip or glenohumeral joints, or biceps tenosynovitis (4). Similar findings may be seen on FDG PET-CT (3,5); PET carries the additional benefit of excluding concomitant large vessel vasculitis or malignancy. However, PET-CT is a scarce resource (our local PET-CT centre is 15 miles away from our own hospital), and carries a significant radiation burden, especially when being considered in younger patients. Whole-body MRI is more rarely used, but can demonstrate inflammation at the greater trochanter, ischial tuberosity or pubic symphysis (6). Inter-observer variation may be less in MRI than in USS, but availability and cost limit its use in some centres (7).

Glucocorticoids remain the cornerstone of PMR treatment, with most patients responding to 12.5–25 mg of prednisolone daily (8). Current EULAR/ACR guidance recommends avoiding initial prednisolone doses exceeding 30 mg/day, as failure to respond to standard doses should prompt reconsideration of the diagnosis rather than dose escalation (8).

Methotrexate is recommended as an adjunct to glucocorticoids in patients at high risk of relapse or prolonged steroid exposure, although evidence supporting its efficacy remains inconsistent (9). More recently, interleukin-6 receptor inhibitors, including tocilizumab and sarilumab, have demonstrated encouraging results in clinical trials for refractory PMR (10,11,12,13). At present, they are not approved for PMR treatment in the UK (1,8).

**Key learning points:**

• The diagnosis and management of polymyalgia rheumatica (PMR) remain challenging because of its non-specific clinical features, lack of disease-specific biomarkers, and limited role of established imaging modalities. As demonstrated in this case, a broad range of inflammatory conditions can mimic PMR, and misdiagnosis may delay appropriate treatment for disorders such as inflammatory arthritis and large vessel vasculitis. In patients with an inadequate response to conventional glucocorticoid therapy, alternative or coexisting diagnoses should be actively reconsidered.

• This case illustrates a presentation of PMR where the diagnosis was uncertain due to the severity of symptoms and large doses of prednisolone needed to provide therapeutic benefit. In cases of diagnostic doubt, a methodical approach should be used to generate a differential diagnosis; investigations can then be used to confirm or refute these differentials. Even then, in this case, the differential was broad, requiring a comprehensive work-up. Inflammatory myopathy and degenerative change were excluded by imaging and immunology. A large vessel vasculitis was considered likely given the high CRP and constitutional upset, but was excluded by PET-CT.

• Even following PET-CT, the patient demonstrated features consistent with both PMR and a seronegative inflammatory arthritis. The former was supported by inflammation in peri-articular tissues; the latter was implied by glenohumeral synovitis on CT-PET, and by the high prednisolone dose required for symptom relief. This made the final diagnosis uncertain but allowed us certain flexibilities with regards to treatment decisions. Given that treatment options are limited in PMR as detailed above, recognising the presence of inflammatory arthritis prompted us to consider the use of tocilizumab as an effective steroid-sparing agent.

